# A Novel Collision-Free Homotopy Path Planning for Planar Robotic Arms

**DOI:** 10.3390/s22114022

**Published:** 2022-05-26

**Authors:** Gerardo C. Velez-Lopez, Hector Vazquez-Leal, Luis Hernandez-Martinez, Arturo Sarmiento-Reyes, Gerardo Diaz-Arango, Jesus Huerta-Chua, Hector D. Rico-Aniles, Victor M. Jimenez-Fernandez

**Affiliations:** 1 Electronics Department, National Institute for Astrophysics, Optics and Electronics, Luis Enrique Erro 1, Santa María Tonantzintla, Cholula 72840, Puebla, Mexico; gcvelez@inaoep.mx (G.C.V.-L.); luish@inaoep.mx (L.H.-M.); jarocho@inaoep.mx (A.S.-R.); 2 Facultad de Instrumentacion Electronica, Universidad Veracruzana, Cto. Gonzalo Aguirre Beltran S/N, Xalapa 91000, Veracruz, Mexico; vicjimenez@uv.mx; 3 Consejo Veracruzano de Investigacion Cientifica y Desarrollo Tecnologico (COVEICYDET), Av. Rafael Murillo Vidal No. 1735, Xalapa 91069, Veracruz, Mexico; 4 Instituto Tecnologico Superior de Poza Rica, Tecnologico Nacional de Mexico, Luis Donaldo Colosio Murrieta S/N, Poza Rica 93230, Veracruz, Mexico; gerardo.diaz@itspozarica.edu.mx (G.D.-A.); chua@itspozarica.edu.mx (J.H.-C.); 5 Electrical Engineering Department, North Central College, 30 N. Brainard St., Naperville, IL 60540, USA; hdricoaniles@noctrl.edu

**Keywords:** collision-free path planning, autonomous robot, robot arm, homotopy continuation methods

## Abstract

Achieving the smart motion of any autonomous or semi-autonomous robot requires an efficient algorithm to determine a feasible collision-free path. In this paper, a novel collision-free path homotopy-based path-planning algorithm applied to planar robotic arms is presented. The algorithm utilizes homotopy continuation methods (HCMs) to solve the non-linear algebraic equations system (NAES) that models the robot’s workspace. The method was validated with three case studies with robotic arms in different configurations. For the first case, a robot arm with three links must enter a narrow corridor with two obstacles. For the second case, a six-link robot arm with a gripper is required to take an object inside a narrow corridor with two obstacles. For the third case, a twenty-link arm must take an object inside a maze-like environment. These case studies validated, by simulation, the versatility and capacity of the proposed path-planning algorithm. The results show that the CPU time is dozens of milliseconds with a memory consumption less than 4.5 kB for the first two cases. For the third case, the CPU time is around 2.7 s and the memory consumption around 18 kB. Finally, the method’s performance was further validated using the industrial robot arm CRS CataLyst-5 by Thermo Electron.

## 1. Introduction

Significant advances have been made in robotics with more powerful and versatile robots being developed. Currently, robots exhibit enhanced capabilities for performing autonomous and semi-autonomous tasks with a high degree of human interaction. Their applications have been expanded from the traditional ones used in industry and research environments to areas such as clinical surgery and rehabilitation therapy [1,2,3]. Moreover, the robotics field offer support functions such as automated navigation, warehouse management, and household management [2,4,5,6,7,8,9].

The design of robotic arms has been part of the robotics evolution by incorporating not only new materials and mechanical structures, but also novel application-specific models. An example of this evolution is hyper-redundant robots; these have a mechanical structure capable of deforming continuously according to their degrees of freedom (DoF) to adapt to disorderly (unstructured) environments [10,11]; these robots resemble living organisms or their parts, such as snakes or elephant trunks; they are denoted as continuous manipulators and are widely applied in the medical area (for minimal invasive surgeries), for in-orbit servicing, grasping, and locomotion in unstructured environments [5,12,13]. However, working with this type of robot implies solving complex and computationally costly inverse kinematics and real-time collision-free path planning problems [10,11,14].

To perform its main task, every robot executes a sequence of movements. This action must be achieved safely, i.e., without colliding with any obstacle in the workspace. Commonly, this task is performed by a planning algorithm responsible for determining a collision-free path that allows the motion of the robot from an initial to a final configuration without colliding with any obstacle. Since obstacles may be static or moving, the algorithm may be recast as an off-line or on-line planner. On-line planning strategies generate the path to the goal during the movement, while off-line planners obtain the path to the goal before the movement begins [15].

**Figure 1:**
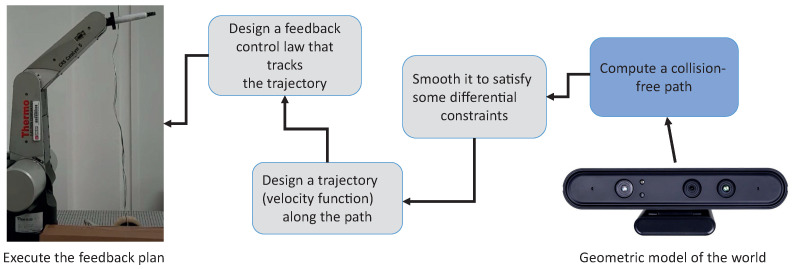
Refinement approach for autonomous robotic manipulators.

Figure 1 shows the stages required for autonomous or semi-autonomous movement in robots [16]. The proposed work contributes to the advancement of collision-free path computations, represented in the blue block.

In the last few decades, several algorithm have been used to determine collision-free paths, and some of the most common are as follows: (a) sampling-based planning algorithms such as rapidly random trees (RRT) [16], probabilistic roadmap (PRM) [1,17], and the variants of each of them [16]; (b) graph-based algorithms such as visibility graph [18] and A* [19]; (c) heuristic-based algorithms such as ant colony [20] and genetic-based [3]; (d) deterministic-based methods, which include artificial potential fields (APFs) [21] and the homotopy-based path-planning method (HPPM) [22,23,24]. These algorithms and methods have been applied in mobile terrestrial robots, UAVs, car-like vehicles, and robotic manipulators [1,16,17,20,23,25,26,27,28]. However, these algorithms and methods still have several drawbacks such as falling into local minima, high computational cost, or long times to obtain a solution path, and some of these do not guarantee a solution path. Some algorithms present difficulties working in complex environments with narrow corridors, a high number of obstacles, or a high number of DoFs. Other path-planning algorithms need a post-processing stage to smooth out the obtained path for implementation in a real robot [16,23,29,30].

Path planning for planar robots is an interesting topic in robotics due to its principle of operation. It is at the root of multi-joint serial manipulator’s path planning because many industrial robots operate with this configuration, such as the SCARA robots. Recently, some works have focused on strategies to handle collision-free path planning. In [31], a path-planning method based on an obstacle-free workspace was proposed for collision-free movements of multi-joint serial manipulators. The method presented in this work uses Monte Carlo and rapidly exploring random tree (RRT), which has advantages in solving the planning problem of a three-joint planar robot arm with only one circular obstacle. Amit Jena et al. proposed in [32] an optimal planning technique using geodesics to achieve an accurate, as well as smooth trajectory for industrial robot manipulators. In this work, the authors validated its technique with a real SCARA robot; however, the workspace in the case study was obstacle-free. In [33], an optimum trajectory planning for a planar redundant manipulator was presented by minimizing the power consumption when its end-effector was commanded to move in its prescribed path. In this work, a model of the three-joint planar arm was used to validate the effectiveness of the methodology. The case studies in this work show the advantages of the planner to minimize the power consumption; however, only one polygonal obstacle is embedded in the workspace. Finally, a method for trajectory planning and control of planar robots with a passive rotational last joint was presented in [34]. This work validated the method for trajectory planning and control problems for the class of n-link planar robots with a passive rotational last joint. The case studies presented in this work were focused on showing the advantages of this method for trajectory planning and control; however, the presence of obstacles in the environment was not considered.

In this work, the operation principle of the path planning homotopic method (HPPM) is modified to deal with the planar robotic arm’s constraints. Originally, the homotopy of continuation was formulated with the aim of solving non-linear systems of equations with multiple solutions. These systems are common in all areas that involve non-linear modeling of systems, such as physics, chemistry, electronics, robotics, fluid mechanics, etc. [23,24,35,36,37,38,39]. Although homotopy has proven to be a very useful tool to find a solution in non-linear systems, recently, its application has focused on solving inverse kinematics calculations in parallel manipulators [35,40], in the calculation of path planning in mobile robots [23,24], and path planning based on the homotopy class with restrictions [41].

In this work, the novel use of the homotopic continuation method for collision-free path planning for planar robotic arms is presented. This involves the modeling of the planar robotic arm and the obstacles in the homotopic formulation to generate the collision-free path. The obtained path is expressed as a sequence of movements for the robotic to move from position A to position B while avoiding obstacles. The paper is organized as follows. Section 2 presents the homotopy-based path planning formulation. The proposed method applied to planar robotic arms with obstacles is presented in Section 3 and Section 4. Three case studies show the performance of the proposed method in Section 5. Section 6 presents a scenario where the proposed method is validated in an industrial robotic arm. Finally, Section 7 presents conclusions and future work.

## 2. Path Planning Using Homotopy-Based Formulations

The homotopy-based path-planning method (HPPM) assumes that the environment constrainsthe robot, and the obstacles present are mathematically modeled and contained in a non-linear algebraic equations system (NAES) represented by (1).

(1) $$F\left(X\right)=0;\;R^{n}⟶R^{n},$$

The use of a homotopy continuation method (HCM) modifies the NAES by introducing an additional parameter known as the homotopy parameter λ, yielding a set of homotopy formulas, expressed by (2).

(2) H(F(X),λ)=λF(X)+(1−λ)G(X)=0,

where $H\left(F\left(X\right),\lambda \right):R^{n+1}⟶R^{n},\;X\in R^{n},\;\lambda \in \left[0,1\right]$ and G(X)=0 is a function with a trivial or known solution. When λ=0, the system in (2) reduces itself to:

(3) $${H\left(F\left(X\right),\lambda \right)|}_{\lambda =0}=G\left(X\right)=0,$$

whereas at λ=1, the system in (2) becomes:

(4) $${H\left(F\left(X\right),\lambda \right)|}_{\lambda =1}=F\left(X\right)=0,$$

Equation (4) becomes the original equation system represented in (1).

A particular case of HCM arises if $G\left(X\right)=F\left(X\right)-F\left(X_{0}\right)$ in (2), which yields (5).

(5) $$H\left(F\left(X\right),\lambda \right)=F\left(X\right)-\left(1-\lambda \right)F\left(X_{0}\right)=0,$$

where $X_{0}$ is the initial point. The formulation in (5) is known as Newton’s homotopy, and it has been used to determine a collision-free path, namely the homotopy path-planning method (HPPM) [22,23].

The HPPM models the robot and the obstacles in the workspace as a set of non-linear algebraic equations. Singularities are created for obstacles so that they can be avoided [22,24]. This yields a system of non-linear equations that is solved by the HCM. The solution curve of H(F(X),λ), is used as the path that the robot will travel from the initial position to the final. An enhanced version of the HPPM (EHPPM) allows better control over the repulsion effect that is caused by the singularities placed in the workspace [22,23,24].

**Figure 2:**
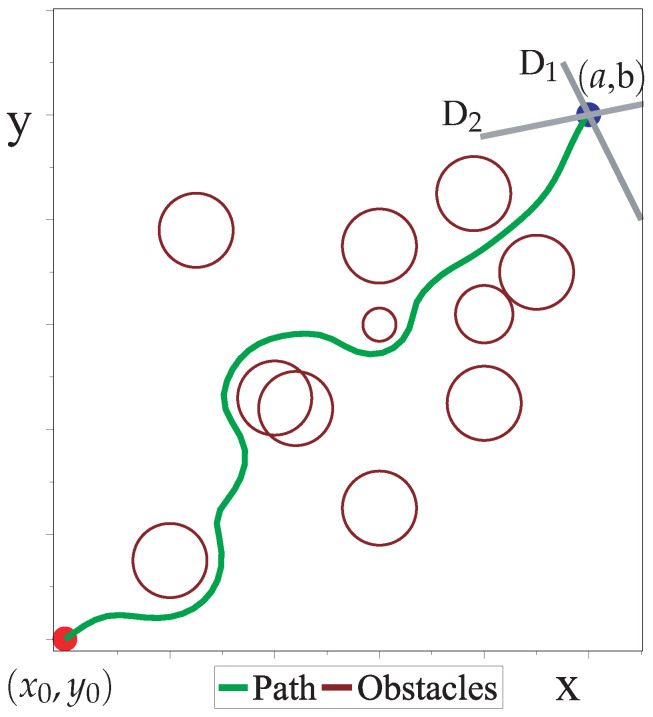
Path obtained with the EHPPM for a mobile robot.

The HPPM has been successfully applied to mobile robots [23,24]. Figure 2 shows the application of the EHPPM to generate a collision-free path for a mobile robot, showing a workspace with a mobile robot and 11 circular obstacles. The initial position of the robot is at $\left(x_{0},y_{0}\right)$ (red point), and the goal is at (a,b) (blue point), while the path to be followed by the robot is shown in blue.

The EHPPM uses the auxiliary Equations (6) and (7) to find the solution.

(6) $$D_{1}\left(x,y\right)=-y-m_{1}x+\left(b+m_{1}a\right)=0,$$

(7) $$D_{2}\left(x,y\right)=-y-m_{2}x+\left(b+m_{2}a\right)=0,$$

where $D_{1}$ and $D_{2}$ are straight lines that intersect at the goal (gray lines in Figure 2), $m_{1}$ and $m_{2}$ are the values of their slopes, and (a,b) represents the ordered pair of the point at the intersection of the straight lines [22,23,24].

The EHPPM models the workspace with Equations (8) and (9):

(8) $$f_{1}\left(x,y\right)=D_{1}\left(x,y\right)=0,$$

(9) $$f_{2}\left(x,y\right)=D_{2}\left(x,y\right)+W\left(x,y\right)-Q=0,$$

where W(x,y) represents the singularities that model the obstacles in the workspace and Q=W(a,b) removes the effect of singularities at the goal point (a,b) of the workspace [22,23,24]. When applying Newton’s homotopy (5), the system of homotopic equations is (10).

(10) $$H=\left\{\begin{array}{c} H_{1}\left(f_{1}\left(x,y\right),\lambda \right)=f_{1}\left(x,y\right)-\left(1-\lambda \right)f_{1}\left(x_{0},y_{0}\right)=0,\\ H_{2}\left(f_{2}\left(x,y\right),\lambda \right)=f_{2}\left(x,y\right)-\left(1-\lambda \right)f_{2}\left(x_{0},y_{0}\right)=0, \end{array}\right.$$

where ($x_{0},y_{0}$) is the start point.

The EHPPM can model the obstacles by two separate sets, namely a set of circles (circular obstacles) and another set of ellipsoidal approximations (ellipsoidal obstacles) [22,23,24]. As a result, W(x,y) can be represented by Equation (11).

(11) $$W\left(x,y\right)=W_{C}\left(x,y\right)+W_{R}\left(x,y\right),$$

where $W_{C}\left(x,y\right)$ represents the set of circular obstacles and $W_{R}\left(x,y\right)$ represents the set of ellipsoidal obstacles.

On the one hand, the set of circular obstacles is defined by Equation (12).

(12) $$W_{C}\left(x,y\right)=\sum_{i=1}^{i=k}\frac{PC_{i}}{C_{i}\left(x_{i},y_{i}\right)},$$

where $PC_{i}$ is the repulsion parameter of each obstacle, *k* is the number of circular obstacles present in the workspace, and $C_{i}\left(x,y\right)$ represents a circular obstacle, modeled by Equation (13).

(13) $$C_{i}\left(x,y\right)={\left(x-x_{i}\right)}^{2}+{\left(y-y_{i}\right)}^{2}-rc_{i}^{2}=0,$$

where $\left(x_{i},y_{i}\right)$ is the center and $rc_{i}$ is the radius of the *i*-th circle.

On the other hand, the set of ellipsoidal obstacles is modeled by Equation (14).

(14) $$W_{R}\left(x,y\right)=\sum_{j=1}^{j=d}\frac{PR_{j}}{R_{j}\left(x_{j},y_{j}\right)},$$

where $PR_{j}$ is the repulsion parameter of each ellipsoidal obstacle, *d* is the number of ellipsoidal obstacles present in the workspace, and $R_{j}\left(x,y\right)$ represents the ellipsoidal obstacle modeled by the equation of an ellipse expressed in (15).

(15) $$R_{j}\left(x,y\right)={\left(\frac{x-x_{j}}{\alpha_{j}}\right)}^{2\eta }+{\left(\frac{y-y_{j}}{\beta_{j}}\right)}^{2\eta }-1=0,$$

where $\left(x_{j},y_{j}\right)$ is the center of the *j*-th ellipsoidal obstacle, $\alpha_{j}$ and $\beta_{j}$ define the width and length, and η is an integer and defines the sharp of the vertex (in this work, η=2).

### 2.1. Spherical-Path-Tracking Algorithm

**Figure 3:**
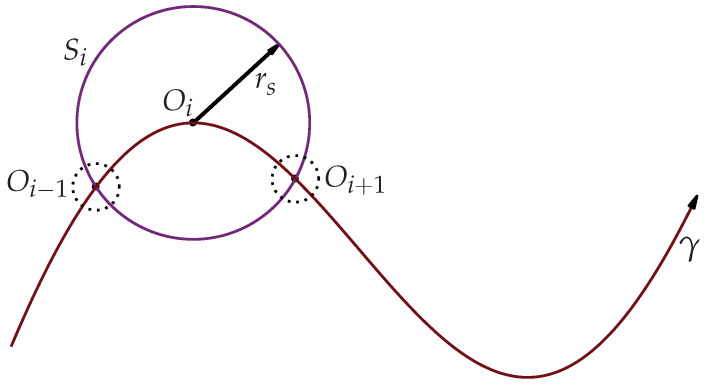
Representation of a hypersphere on the homotopic curve γ.

The EHPPM uses the spherical tracking algorithm to follow the homotopy path at all times and avoids falling into discontinuities or closed curves [22,23,24,42]. This algorithm creates a hypersphere *S* of *n* dimensions, where the number of dimensions corresponds to the number of generated homotopic equations. The hypersphere $S_{i}$ has a radius $r_{s}$ with center $O_{i}$ located on the homotopic curve γ. The circumference of the sphere touches at least two points $\left(O_{i-1},O_{i+1}\right)$ on the curve γ, as depicted in Figure 3.

Equation (16) describes the hypersphere for two homotopy functions.

(16) $$S_{i}\left(x,y,\lambda \right)={\left(x-c_{x}\right)}^{2}+{\left(y-c_{y}\right)}^{2}+{\left(\lambda -c_{\lambda }\right)}^{2}-r_{s}^{2}=0,$$

where $\left(c_{x},c_{y},c_{\lambda }\right)$ is the center and $r_{s}$ is the radius of the hypersphere at each step of the spherical tracking.

Using Newton’s homotopy (5), with Equations (10) and (16), the system of homotopy equations is expressed as:

(17) $$H_{S}=\left\{\begin{array}{c} H_{1}\left(x,y,\lambda \right)=0,\\ H_{2}\left(x,y,\lambda \right)=0,\\ S_{i}\left(x,y,\lambda \right)=0 \end{array}\right.$$

### 2.2. Predictor–Corrector Algorithm

**Figure 4:**
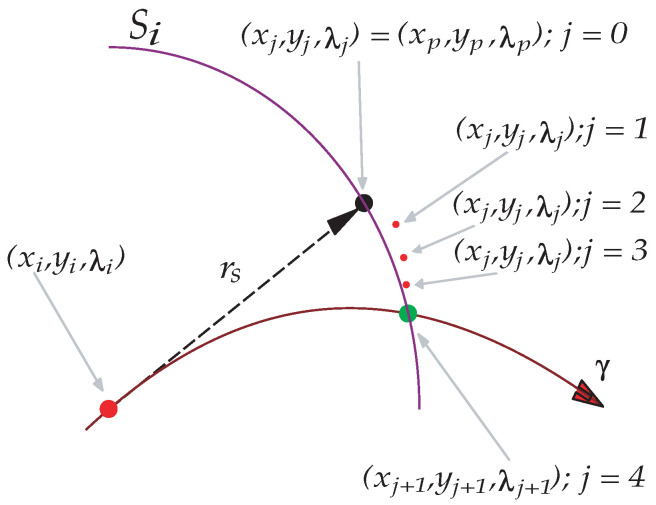
Representation of the predictor–corrector algorithm.

The spherical path tracking of trajectories is complemented by the predictor–corrector algorithm, which helps follow the homotopic path without falling into discontinuities [22,23,24,42]. The predictor algorithm is used to generate the next point close to the homotopic trajectory γ such that the intersection between the hypersphere and the y curve is achieved; in this work, the corrector was implemented with Broyden’s method [24,43], and the intersection between the hypersphere and the γ curve is achieved. Figure 4 shows the predictor–corrector algorithm where $\left(x_{i},y_{i},\lambda_{i}\right)$ is the center of the hypersphere $S_{i}$ and $r_{s}$ represents the radius of the hypersphere. The predictor is the point $\left(x_{j},y_{j},\lambda_{j}\right)=\left(x_{p},y_{p},\lambda_{p}\right)$, and it is used as the starting point for the corrector, finding the intersection of the hypersphere with the curve γ at j=4. Now, the next center of the hypersphere is at $\left(x_{j+1},y_{j+1},\lambda_{j+1}\right)$.

This algorithm repeats and updates the center of each hypersphere until it reaches γ=1. In this work, two predictive methods were implemented: Euler’s predictor [23,24] and the vector predictor [42]. To start the homotopic path, Euler’s predictor is used (only two hyperspheres), then the vector predictor is the one that continues with the path until reaching λ=1; this is due to two reasons: by its formulation, the vector predictor always advances on the curve γ, and it requires a lower computational cost than Euler’s predictor.

## 3. Proposed Scheme for Path Planning of Planar Arms

In this paper, a modified HPPM [22] is presented to obtain a collision-free path for planar robot arms. The proposal leaves aside the holonomic punctual robot and presents a novel scheme that has the ability to model redundant and hyper-redundant rigid planar robotic arms, with or without grippers, providing them with rigidity and orientation, and through the formulation of singular projections implemented, it guarantees the avoidance of obstacles. The proposed method is named the homotopy path-planning method for planar robotic arms (HPPM-PRA).

**Figure 5:**
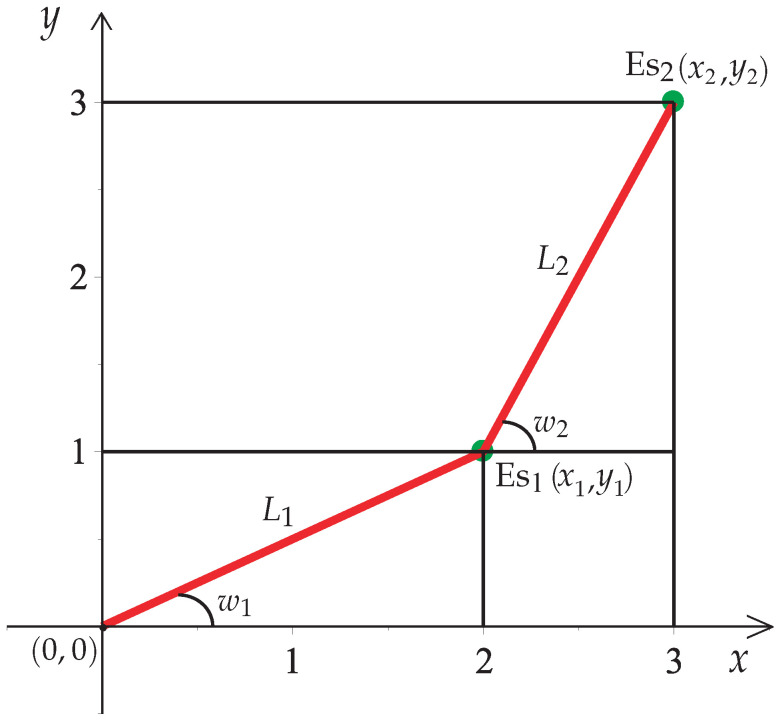
Representation of a two-link planar robot arm.

The inputs of the HPPM-PRA are the initial and final configuration of the planar robotic arm and the obstacle’s position and size. The planar robotic arm configuration is described in terms of the angles of the links, as shown in Figure 5.

The HPPM-PRA works on the configuration space (C-space), and in the proposed method, it is shown with the angles *w* of the bonds; therefore, the algebraic equations are described using *w*-angles, as well as the obstacles. To describe the obstacles in the C-space, it is proposed to project the obstacles from the Euclidean space (x,y) to the C-space, by creating singularities just in the points where the links touch the perimeter of the obstacles. Therefore, the homotopy path avoids obstacles in the C-space.

Based on Figure 5, the formulas that determine the position of a two-link planar robot arm anchored to the point of origin (0,0) are described by (18).

(18) $$\left.\begin{array}{c} {\mathrm{Es}}_{1}:\;\left(x_{1},y_{1}\right)=\left(L_{1}\cos\left(w_{1}\right),L_{1}\sin\left(w_{1}\right)\right),\\ {\mathrm{Es}}_{2}:\;\left(x_{2},y_{2}\right)=\left(x_{1}+L_{2}\cos\left(w_{2}\right),y_{1}+L_{2}\sin\left(w_{2}\right)\right), \end{array}\right.$$

where $L_{1}$ and $L_{2}$ represent the length of the links and the angles $w_{1}$ and $w_{2}$ represent the position of links ${\mathrm{Es}}_{1}$ and ${\mathrm{Es}}_{2}$. This process can be repeated for each link in the case of more than two links.

**Figure 6:**
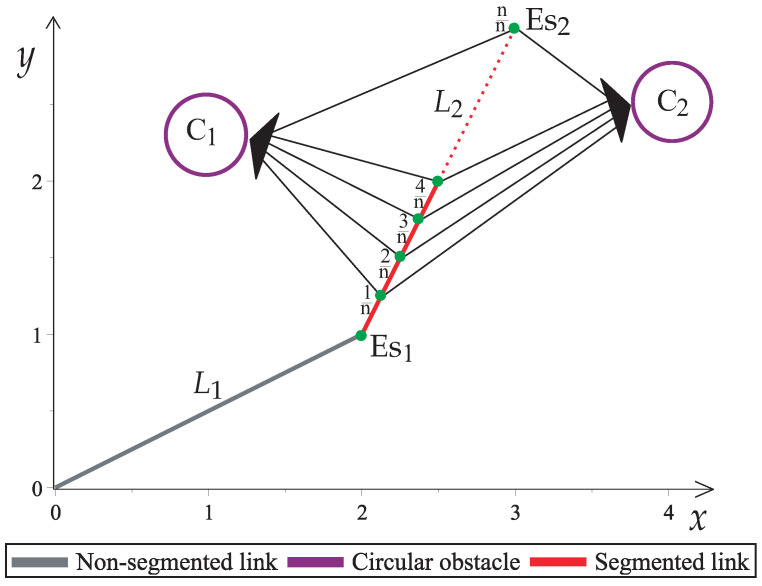
Singular projections of a two-link planar robot arm with two obstacles.

In order to avoid obstacles, each link has to be divided into n singular points. These singular points exhibit a projection over the perimeter of each of the obstacles. Figure 6 shows an example of a workspace with a two-link robot arm (${\mathrm{Es}}_{1},{\mathrm{Es}}_{2}$) and two circular obstacles $\left(C_{1},C_{2}\right)$. The link ${\mathrm{Es}}_{2}$ is divided into *n* singular points. Then, each singular point of Es${}_{2}$ is represented by (19).

(19) $$\begin{array}{c} {\mathrm{Es}}_{2}:\;\left(x_{k},y_{k}\right)=\left(L_{1}\cos\left(w_{1}\right)+\left(q_{k}\right)L_{2}\cos\left(w_{2}\right),L_{1}\sin\left(w_{1}\right)+\left(q_{k}\right)L_{2}\sin\left(w_{2}\right)\right),\\ \;k=1,\ldots ,n, \end{array}$$

where $q_{k}$ helps calculate the position of each singular *k*-th point in the Euclidean space and represents the link’s segments, as represented by Equation (20).

(20) $$q_{k}=\frac{k}{n},\;k=1,2,\ldots ,n,$$

where *k* is the *k*-th segment and *n* is the number of segments.

The formulation for circular obstacles $C_{i}\left(w\right)$ and ellipsoidal obstacles $R_{j}\left(w\right)$ is expressed in (21) and (22).

(21) $$C_{i}\left(w_{1},w_{2}\right)={\left(x_{k}-x_{i}\right)}^{2}+{\left(y_{k}-y_{i}\right)}^{2}-r_{i}^{2},\;i=1,2,\ldots ,c,$$

(22) $$R_{j}\left(w_{1},w_{2}\right)={\left(\frac{x_{k}-x_{j}}{\alpha_{j}}\right)}^{2\eta }+{\left(\frac{y_{k}-y_{j}}{\beta_{j}}\right)}^{2\eta }-1,\;j=1,2,\ldots ,d,$$

where $C_{i}$ is the *i*-th circular obstacle, $R_{j}$ represents the *j*-th ellipsoidal obstacle, *c* is the number of circular obstacles, and *d* is the number of ellipsoidal obstacles in the workspace. The coordinates of each singular point in each link are represented by $\left(x_{k},y_{k}\right)$, while $\left(x_{i},y_{i}\right)$ is the center of circular obstacle ($C_{i}\left(w_{1},w_{2}\right)$) and $\left(x_{j},y_{j}\right)$ is the center of ellipsoidal obstacle ($R_{j}\left(w_{1},w_{2}\right)$). $r_{i}$ is the radius of each circular obstacle, and $\alpha_{j}$ and $\beta_{j}$ are the base and height of the *j*-th ellipsoidal obstacle. To create rigid obstacles, *n*-singular points were used for each link by considering the size of the smallest obstacle in the workspace as a reference to set *n*. The formulation that represents the singularities created by the singular projections involving all the links is given by (23).

(23) $$W\left(w_{1},\ldots ,w_{v}\right)=\sum_{i=1}^{c}\sum_{t=1}^{v}\sum_{k=1}^{n}\frac{P_{Ci}}{C_{i}\left(w_{1},\ldots ,w_{v}\right)}+\sum_{j=1}^{d}\sum_{t=1}^{v}\sum_{k=1}^{n}\frac{P_{Rj}}{R_{j}\left(w_{1},\ldots ,w_{v}\right)},$$

where $P_{Ci}$ represents the repulsion parameter of each circular obstacle, $P_{Rj}$ represents the repulsion parameter of each ellipsoidal obstacle, *c* represents the number of circular obstacles, *d* represents the number of ellipsoidal obstacles, *v* the number of links, and *n* the number of singular points of each link.

The HPPM-PRA uses a system of auxiliary equations to set the final position of the robotic arm given by (24).

(24) $$l_{k}\left(w_{1},\ldots ,w_{v}\right)=a_{0,k}+\sum_{j=1}^{v}a_{j,k}w_{j}=0,\;k=\left[1,2,\ldots ,v\right],$$

Here, *v* is the number of links in the robot arm and *a* is a set of arbitrary constants. By evaluating (24) at the goal position $\left(w_{goal_{1}},\ldots ,w_{goal_{v}}\right)$ and selecting $a_{0,k}$ to set each linear equation to zero, the system of equations for a planar robot arm becomes:

(25) $$\begin{array}{c} f_{1}\left(w_{1},\ldots ,w_{v}\right)=l_{1}\left(w_{1},\ldots ,w_{v}\right)=0,\\ f_{2}\left(w_{1},\ldots ,w_{v}\right)=l_{2}\left(w_{1},\ldots ,w_{v}\right)=0,\\ \vdots \\ f_{v}\left(w_{1},\ldots ,w_{v}\right)=l_{v}\left(w_{1},\ldots ,w_{v}\right)+W\left(w_{1},\ldots ,w_{v}\right)-Q=0, \end{array}$$

where *Q* is a constant to guarantee that the solution of (25) is $\left(w_{goal_{1}},\ldots ,w_{goal_{v}}\right)$. It is important to note that the equation $l_{v}$ was chosen to add the term W−Q; nonetheless, it is feasible to select another equation of (25).

The homotopy system to solve (25) using (5) is then (26).

(26) $$H=\left\{\begin{array}{c} \begin{array}{c} H_{1}\left(f_{1}\left(w_{1},\ldots ,w_{v}\right),\lambda \right)=f_{1}\left(w_{1},\ldots ,w_{v}\right)-\left(1-\lambda \right)f_{1}\left(w_{start_{1}},\ldots ,w_{start_{v}}\right)=0,\\ H_{2}\left(f_{2}\left(w_{1},\ldots ,w_{v}\right),\lambda \right)=f_{2}\left(w_{1},\ldots ,w_{v}\right)-\left(1-\lambda \right)f_{2}\left(w_{start_{1}},\ldots ,w_{start_{v}}\right)=0,\\ \vdots \\ H_{v}\left(f_{v}\left(w_{1},\ldots ,w_{v}\right),\lambda \right)=f_{v}\left(w_{1},\ldots ,w_{v}\right)-\left(1-\lambda \right)f_{v}\left(w_{start_{1}},\ldots ,w_{start_{v}}\right)=0, \end{array} \end{array}\right.$$

where $\left(w_{start_{1}},\ldots ,w_{start_{v}}\right)$ represents mathematically the starting point for the homotopy path and, physically, the initial position of the robotic arm.

Equation (27) is proposed to implement the hyperspherical path-tracking algorithm that was stated in (16).

(27) $$S_{i}\left(w_{1},\ldots ,w_{v},\lambda \right)={\left(w_{1}-c_{1}\right)}^{2}+{\left(w_{2}-c_{2}\right)}^{2}+\cdots +{\left(w_{v}-c_{v}\right)}^{2}+{\left(\lambda -c_{v+1}\right)}^{2}-r_{s}^{2}=0,$$

where $\left(c_{1},c_{2},\cdots ,c_{v+1}\right)$ is the center of the hypersphere and $r_{s}$ is the radius of the hypersphere. It is important to highlight that the first hypersphere’s center $\left(c_{1},c_{2},\cdots ,c_{v}\right)$ is equal to the starting point $\left(w_{start_{1}},\ldots ,w_{start_{v}}\right)$ of the homotopy at $\lambda =c_{v+1}=0$. However, it is also feasible to use a variable-radius scheme.

The system of equations to be solved for each step of the path-tracking algorithm is given in (28).

(28) $$H_{S}=\left\{\begin{array}{c} \begin{array}{c} H_{1}\left(w_{1},\ldots ,w_{v},\lambda \right)=0,\\ H_{1}\left(w_{1},\ldots ,w_{v},\lambda \right)=0,\\ \vdots \\ H_{v}\left(w_{1},\ldots ,w_{v},\lambda \right)=0,\\ S_{i}\left(w_{1},\ldots ,w_{v},\lambda \right)=0, \end{array} \end{array}\right.$$

where the center of the hypersphere $S_{i}$ is updated at every *i*-th step of the path tracking, as explained in Section 2.2.

### Workspace and C-Space

**Figure 7:**
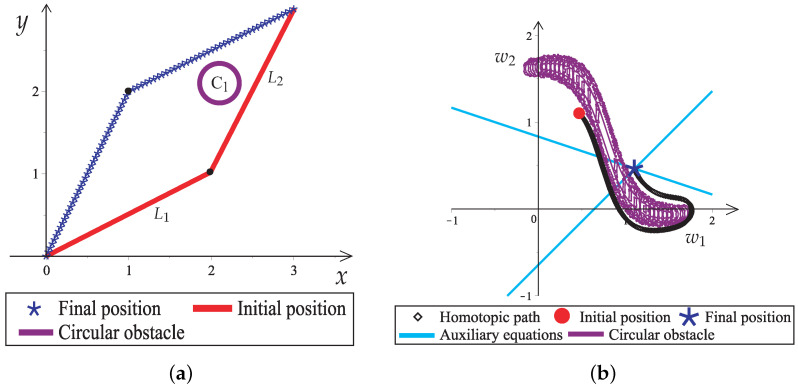
Workspace to C-space of a two-link planar robot arm with a circular obstacle. (**a**) Two-link planar robot workspace. (**b**) C-space representation of the two-link planar robot workspace.

This section explains how to transform from the workspace to C-space and the relation to obstacles and the homotopic path computation process. On the one hand, the workspace is the graphical representation of the number and length of links, the initial and final position of the robotic arm (start, goal), and the number, position, and shape of obstacles in the real world. On the other hand, the C-space maps the allowed and forbidden configurations that the robot can perform without collisions in the workspace. The configurations are modeled in a space with as many dimensions as degrees of freedom the robot has. Figure 7a shows the workspace with a two-link planar robotic arm. The initial (start) and final (goal) positions of the robotic arm are represented by the solid red line and the blue asterisks, respectively. Figure 7b shows the C-space in terms of the angles $w_{1}$ and $w_{2}$ of the links.

The solid red dot marks the starting point (start), and the intersection of the auxiliary lines denotes the endpoint (goal) of the homotopic path (blue diamond).

It can be noted that the circular obstacle in the Euclidean space is transformed into an elongated amorphous shape in the C-space, and this is due to the singular projections that exist between the link of the robotic arm and the obstacle; this shape was obtained by plotting (23) using the Maple 18 “implicitplot” command. It should be mentioned that for more than three dimensions, it is not easy to visualize the C-space.

## 4. HPPM-PRA Procedure Steps

The HPPM-PRA is a straightforward procedure that is easy to implement. This requires some basic steps:

1. Capturing of the workspace. In this step, a camera or system to capture the environment is used to generate the geometrical representation of the robot’s workspace. This procedure requires an image-processing algorithm; however, this is not a topic of this work; thus, the workspace is considered known a priori. Then, for this work, only circular obstacles (21) and ellipsoidal obstacles (22) are considered to represent the workspace in the case studies of Section 5.
2. Setting the robot parameters. The number and length of the links and the start and end configurations are given to the HPPM-PRA.
3. Repulsion parameter assignation. Assign the repulsion parameter to each obstacle of the workspace.
4. Model workspace obstacles in the C-space. The singular projections (*W*) that represent the forbidden configurations (obstacle collision space) are employed to establish the *Q* value using (23).
5. Generate the auxiliary equations. These are used to set the final configuration of the robotic arm.
6. Generate non-linear equation system. This represents the entire problem and contains the characteristics of the robot and the workspace.
7. Homotopy continuation formulation (Equation (26)). In this step, the original system of non-linear equations of the previous step is converted to a homotopic system.
8. Hyperspherical tracking. The hyperspherical tracking algorithm is employed to calculate each point of the solution path.
9. Robotic arm executing. Finally, the obtained homotopic path is followed by the robotic arm.

**Figure 8:**
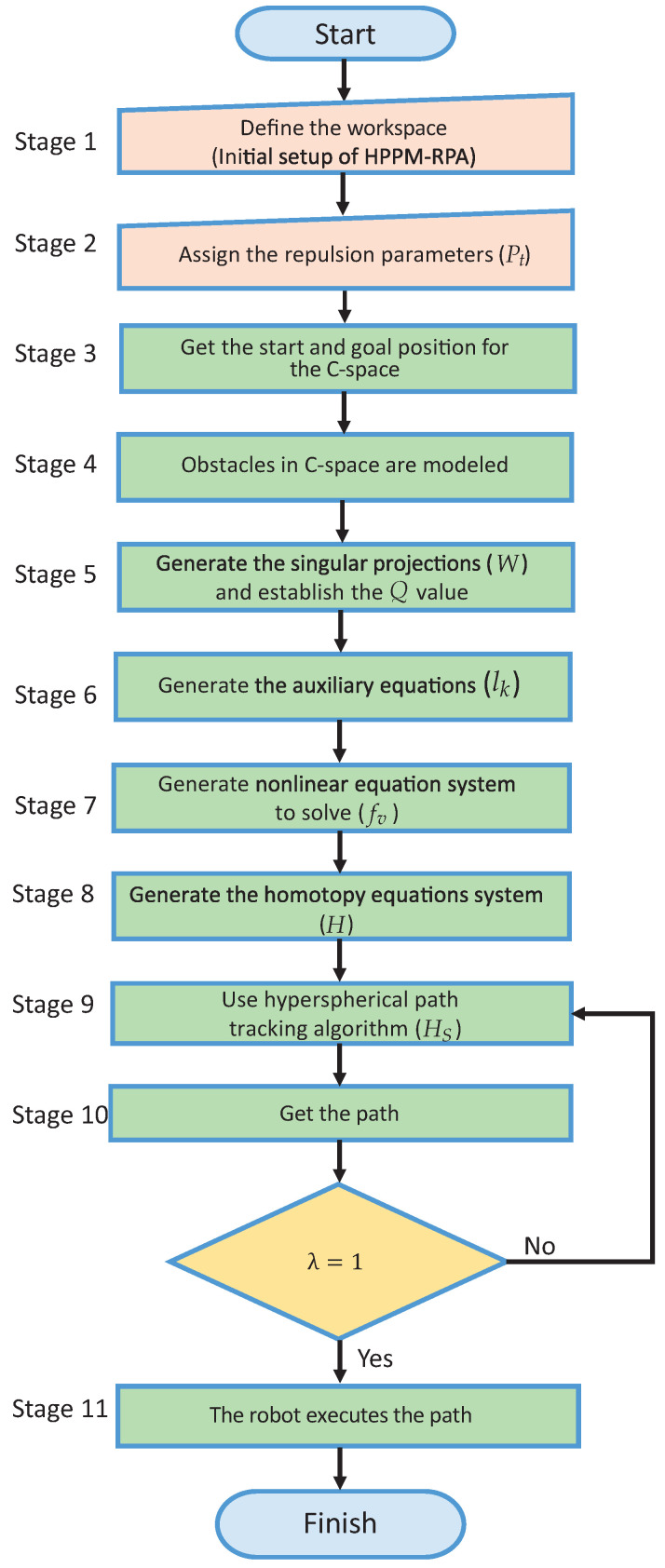
HPPM-PRA method flowchart.

Figure 8 shows the flowchart of the proposed methodology.

Algorithms 1–5 describe the stages that model the proposed method. Algorithm 1 describes the general procedure of the HPPM-PRA. This procedure requires the workspace configuration as the input and uses four algorithms that build the homotopy system (*H*). The homotopy system is solved by using the spherical path-tracking algorithm to generate the path for the planar robotic arm.

The function of Algorithm 2 is to transform the workspace to the C-space, and the information obtained ($w_{start},w_{goal},a_{0}$) is used by the following algorithms.

Algorithm 3 is responsible for obtaining the value of *Q* from the evaluation of the circular and ellipsoidal obstacles in the final position $\left(w_{goal}\right)$. Furthermore, this algorithm is used in each step of the hyperspherical procedure to obtain the value of *W*.

Algorithm 4 builds, based on the auxiliary equations ($l_{k}\left(w_{1},\ldots ,w_{v}\right)$), the system of non-linear equations ($f_{1},\ldots ,f_{v}$), which Algorithm 5 uses to generate the homotopic system (*H*).

**Algorithm 1** HPPM-PRA general procedure.
**Require:**  R(w),C(w), $Es_{start},Es_{goal}$, L,v▹ The task to be solved is proposed**Require:**  $P_{C},P_{R}$▹ Assign the repulsion parameter1: Get $\left(w_{start},w_{goal},a_{0}\right)$▹ See Algorithm 22: Get the value of *Q*▹ See Algorithm 33: Set the non-linear equation system to solve ($f_{1},\ldots ,f_{v}$) ▹ See Algorithm 44: Generate the homotopy equation (*H*) ▹ See Algorithm 55: Create hypersphere $S_{i}$▹$w_{start}$ is used as the center of the first hypersphere6: Formulation of the homotopy system ($H_{S}$)
7: iteration=0 ▹ A temporary variable is used as the counter8: **while**(λ≠1)**do**▹ Use the hyperspherical path-tracking algorithm, until λ=19:     **if** (iteration<2) **then**
10:         Euler’s predictor ($H_{S}$) ▹ Euler’s predictor is used11:     **else**
12:         Vector predictor ($H_{S}$) ▹ The vector predictor is used13:     **end if**
14:     Broyden’s method ($H_{S}$) ▹ The corrector method is used15:     The numeric homotopy path is stored
16:     Update the center of the hypersphere ($S_{i}$)
17:     iteration++
18: **end while**
**Ensure:**  The numerical homotopy path ▹ The robotic arm can execute the path

**Algorithm 2** Transformation from workspace to C-space.
1: **function**C-space(start,goal, $a_{j,k}$, $v,L_{v}$)
2:     j,k▹ Temporary variables3:     **for** (j=0;j<v;j++) **do**
4:         $w_{start}\left[j\right]=\arccos\left(\frac{start[j+1]-start[j]}{L[j]}\right)$▹ Start position in C-space.5:         $w_{goal}\left[j\right]=\arccos\left(\frac{goal[j+1]-goal[j]}{L[j]}\right)$▹ Goal position in C-space.6:     **end for**
7:     **for** (j=0;j<v;j++)**do**▹ Calculation of the value of $a_{0}$.8:         **for** (k=0;k<v;k++) **do**
9:            $a_{0}\left[j\right]=\left(w_{goal}\left[k+1\right]\right)\left(a\left[j\right]\left[k\right]\right)$
10:         **end for**
11:         $a_{0}\left[j\right]-=w_{goal}\left[0\right]$
12:     **end for**
13:     **return** $\left(w_{start},w_{goal},a_{0}\right)$▹ Returns the values from the C-space14: **end function**


**Algorithm 3** Get the value of (*Q*, *W*).
1: **function**Get *Q*($w_{goal}$,$C_{w}$,$R_{w}$,$P_{C}$,$L_{v}$,c,v,n)
2:     $add_{obs}\left[v\right]=0$,add[n]=0,j,k,i▹ Temporal variables.3:     **for** (j=0;j<c;j++) **do**
4:         **for** (k=0;k<v;k++) **do**
5:            **for** (i=0;i<n;i++) **do**
6:                $x_{k}\left[i\right]=\cos\left(w_{goal}\left[k\right]\right)\left(L\left[k\right]\right)\left(\frac{i+1}{n}\right)$
7:                $y_{k}\left[i\right]=\sin\left(w_{goal}\left[k\right]\right)\left(L\left[k\right]\right)\left(\frac{i+1}{n}\right)$▹$\left(x_{k},y_{k}\right)$ are the coordinates of each singular point for a given link8:                $add_{obs}\left[k\right]+=\frac{P_{C}\left[j\right]}{{\left(x_{k}\left[i\right]-x\left[j\right]\right)}^{2}+{\left(y_{k}\left[i\right]-y\left[j\right]\right)}^{2}-{\left(r\left[j\right]\right)}^{{}^{2}}}$▹ The equation of the circular obstacle C(w) can be replaced by the equation of ellipsoidal obstacle R(w)9:            **end for**
10:            $add\left[j\right]+=add_{obs}\left[k\right]$
11:         **end for**
12:         $add_{obs}\left[v\right]=0$▹ Temporal variable is cleared.13:         Q+=add[j]
14:     **end for**
15:     **return** (Q)▹ The value of *Q* is obtained.16: **end function**


**Algorithm 4** Set non-linear equation system to solve.
1: **function**Set $f_{1},\ldots ,f_{v}$($a_{0},a_{j,k},W,Q,v$)
2:     j,k
▹ Temporary variables3:     **for** (j=1;j<v;j++) **do**
4:         **for** (k=1;k<v;k++) **do**
5:            l[k]+=(a[j][k])(w[j])
6:         **end for**
7:         $l\left[k\right]+=a_{0}\left[j\right]$▹ The system of auxiliary equations is obtained $l_{k}\left(w_{1},\ldots ,w_{v}\right)$8:     **end for**
9:     **for** (k=1;k<v−1;k++) **do**
10:         f[k]=l[k]
11:     **end for**
12:     f[k+1]=l[k]+W−Q
13:     **return** *f*▹ Returns $f_{1},\ldots ,f_{v}$14: **end function**


**Algorithm 5** Generate the homotopy system.
1: **function**Generate *H*($w_{start},v,f_{1},\ldots ,f_{v},\lambda$)

2:     **for** (k=1;k<v;k++) **do**
3:         $H\left[k\right]=f\left[k\right]-\left(1-\lambda \right)f\left[k\right]\left(w_{start}\right)$
4:     **end for**5:     **return** *f*▹ Returns $H_{1},\ldots ,H_{v}$6: **end function**


## 5. Case Studies

In this section, three case studies are presented, which show the capacity of the proposed method to obtain the path in planar robot arms. These studies evaluate the performance of the proposed method with the environment, which includes narrow corridors and circular and ellipsoidal obstacles. The implementation can be modified to any number of links, their length, and added grippers. For all the case studies shown in this section, the following color convention is used: circular obstacles ($C_{u}$) are purple; ellipsoidal obstacles ($E_{u}$) are gray; the gripper is brown; the initial position of the robot arm is red; the final position is blue; the homotopic path is shown in black. The proposed method was implemented using the C++ programming language, and the animations were performed in Maple software. All the case studies were executed on a personal computer with a Core i5-4210u@1.7GHz processor and 8 GB of RAM. Here, it is important to note that no special specialized package, library, or hardware was used to help reduce the computing time.

### 5.1. Case Study 1

**Figure 9:**
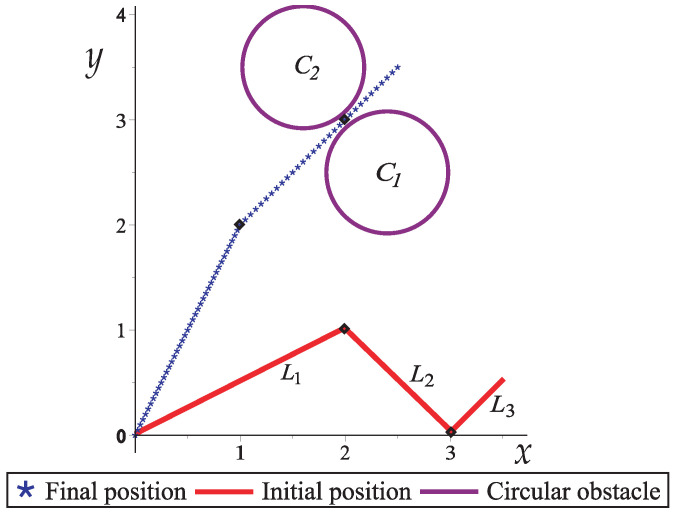
Workspace of a planar robot arm with three links and two circular obstacles.

In this case study, a three-link planar robot arm with two circular obstacles is presented as shown in Figure 9. To reach the final position, the robot arm must pass through a narrow corridor generated by the circular obstacles $C_{1}$ and $C_{2}$.

Table 1 shows the center, radius, and proposed repulsion parameter (*P*) of circular obstacles, the length of each link, and the proposed constants of auxiliary equations.

**Figure 10:**
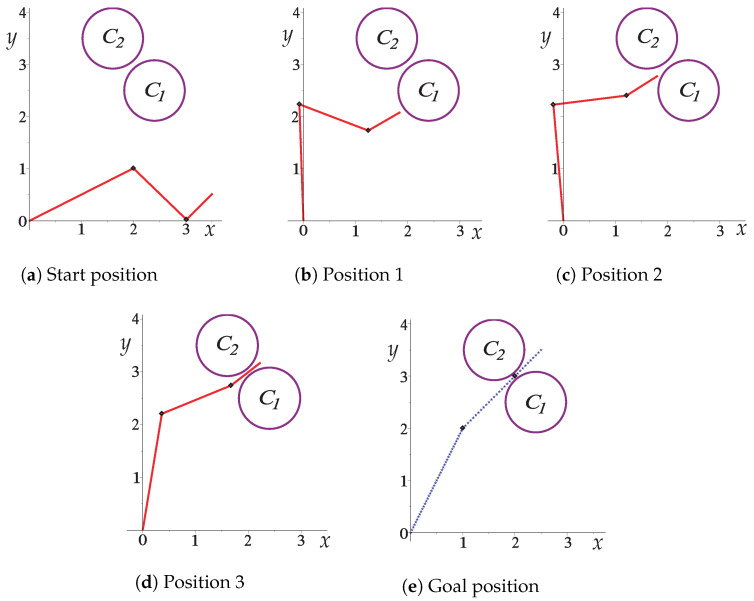
Sequence of images that describe the collision-free path of Case Study 1.

Figure 10 depicts the collision-free path for the robotic arm of Figure 9. From Figure 10a to Figure 10e, the collision-free movements of the planar robotic arm are observed until it reaches the final position (goal) in the narrow corridor generated by the two obstacles (see Figure 10e).

**Figure 11:**
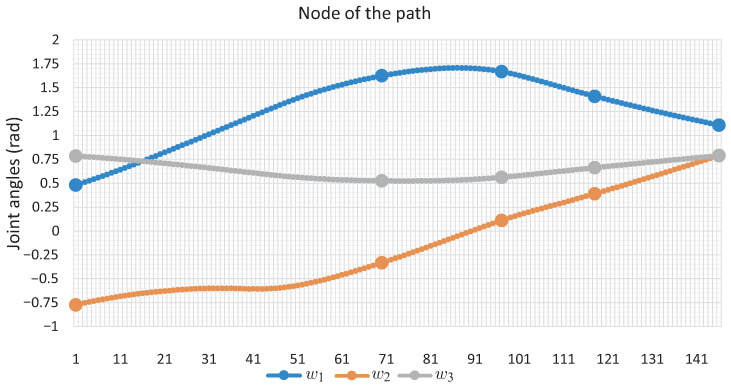
Joint angles motion of Case Study 1.

**Figure 12:**
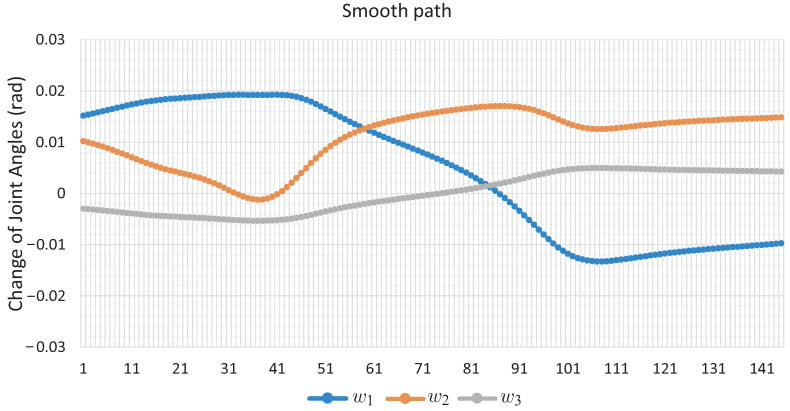
Change of joint angle motion of Case Study 1.

Figure 11 shows the changes that each angle of movement *w* of the robotic arm has; the marked points are the nodes of the solution path shown in Figure 10. Figure 12 shows the change in joint angle movement, where it is observed that the greatest change obtained between each movement does not exceed 0.03 radians, confirming that the path obtained is smooth.

**Figure 13:**
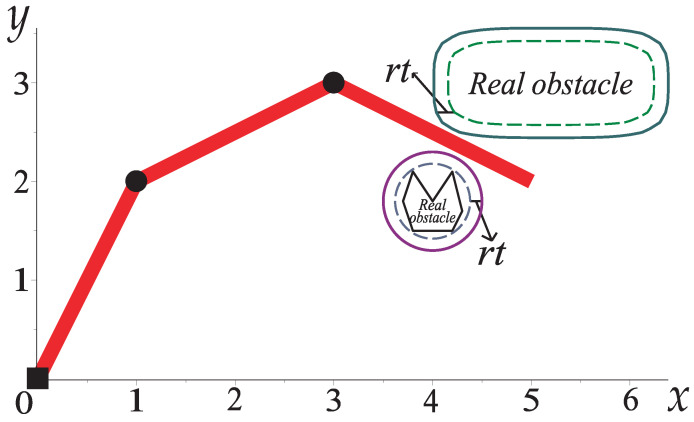
Representation of a safeguard radius (rt) of an obstacle in a workspace with a robotic arm.

It is important to note that the robotic arm touches the perimeter of the obstacles; nevertheless, it does not collide with them due to the safeguard radius (rt) [22,23], as depicted in Figure 13. This case study shows that the HPPM-PRA can obtain paths even in narrow corridors where probabilistic methods may fail.

### 5.2. Case Study 2

**Figure 14:**
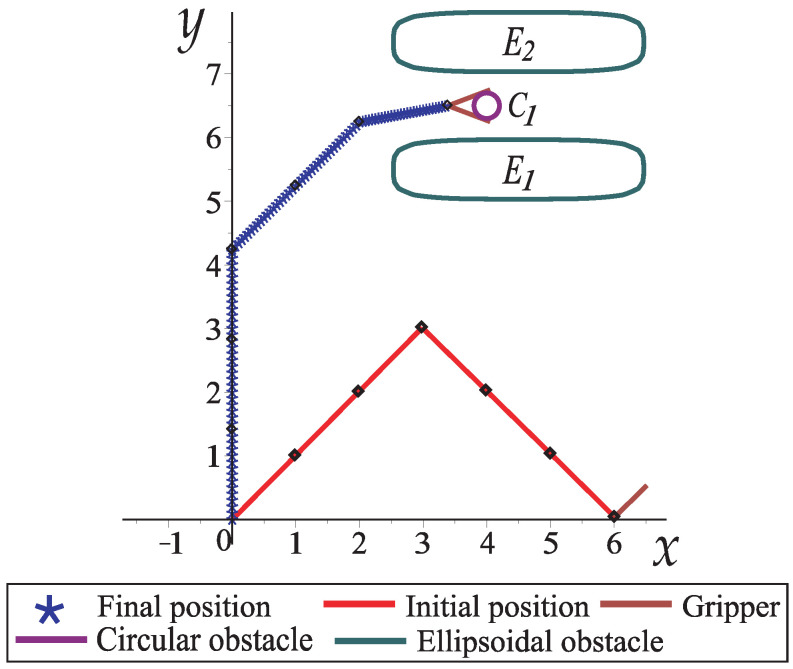
Case Study 2 shows a planar robotic arm with six links and a gripper, two ellipsoidal obstacles, and a circular obstacle.

For this case study, a robot arm with six links (of the same length) and a gripper is depicted. The robotic arm starts from a rest position where the gripper is closed and moves to grip a circular object ($C_{1}$) that is between the ellipsoidal obstacles ($E_{1},E_{2}$), as depicted in Figure 14. Table 2 shows the configuration parameters for this case study.

**Figure 15:**
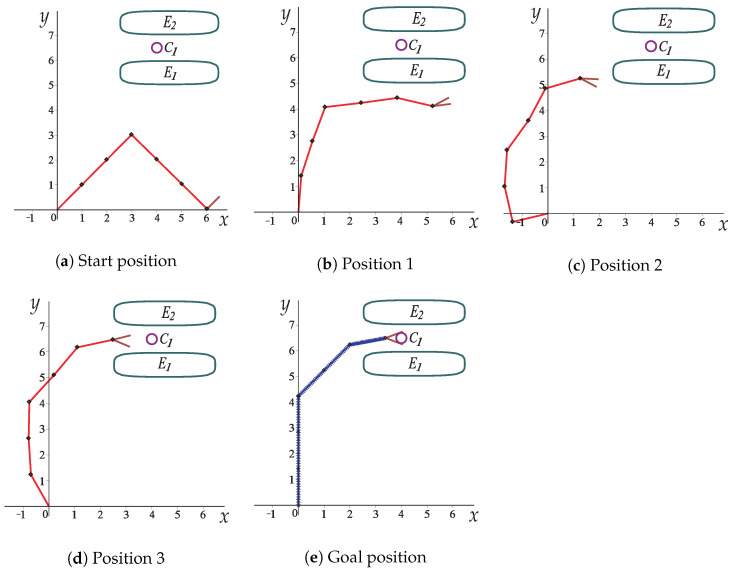
Movement of a six-link planar robot arm with a gripper, obstacles, and a circular object to grip.

Figure 15 shows how the robotic arm moves from the initial position to the final position to grip the circular object. The robotic arm goes through the narrow corridor whilst the gripper opens gradually to hold the object; the HPPM-PRA considers the robotic arm and gripper as a unified structure during simulation. In this case study, a simple gripper was proposed, but it can be modeled in different ways, depending on the specific needs.

**Figure 16:**
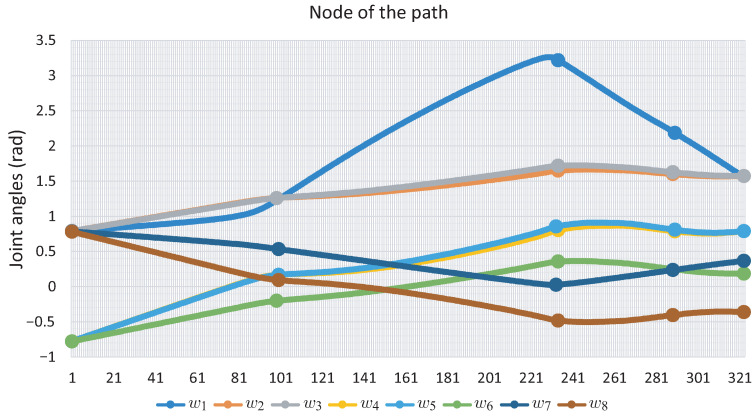
Joint angle motion for Case Study 2.

**Figure 17:**
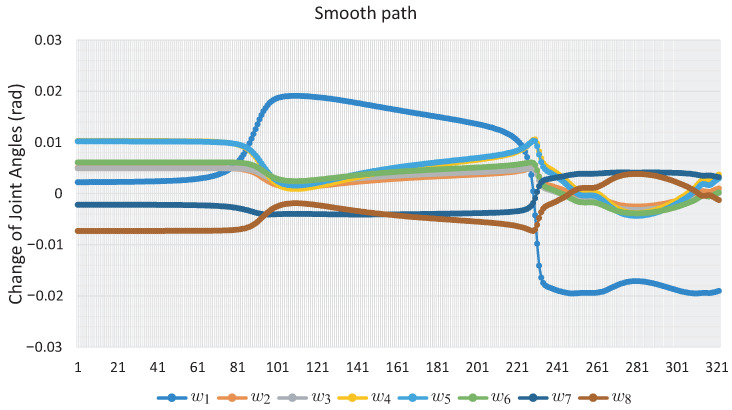
Change of joint angle motion of Case Study 2.

Figure 16 shows the angle *w* of each joint for the full path; the points marked in Figure 16 correspond to the images in Figure 15. Figure 17 shows the change in joint angle movement, where it is observed that the greatest change obtained between each movement does not exceed 0.03 radians for the full path, so the path is smooth.

### 5.3. Case Study 3

**Figure 18:**
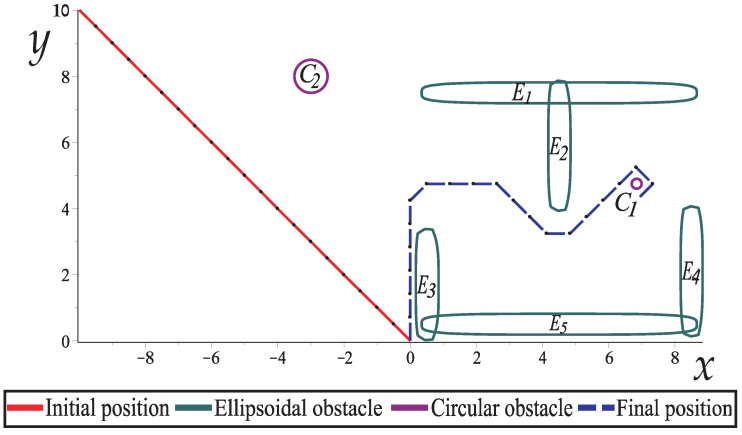
Workspace of a planar robotic arm with twenty link, five ellipsoidal obstacles, and one circular obstacle.

This case study aims to show the suitability of the proposed method applied to a hyper-redundant robot. For this, a twenty-link hyper-redundant robot arm is used. Figure 18 depicts the scenario proposed for this case study. The robot arm must evade the circular obstacle $C_{2}$, continue to move through the path formed by objects $E_{1}$–$E_{5}$, and finally, grab the circular object ($C_{1}$). In this scenario, a gripper is no longer necessary, since the robotic arm can grab the object with its links; this is an advantage of the hyper-redundant arms [10,11].

**Figure 19:**
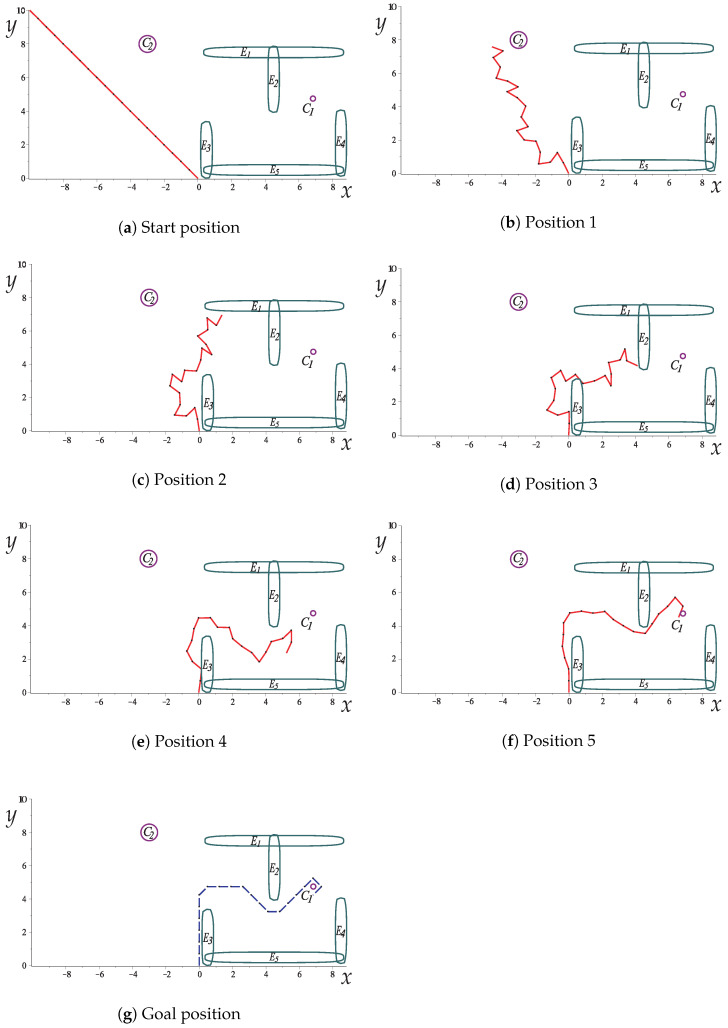
The sequence of images that describe the collision-free path of Case Study 3.

**Figure 20:**
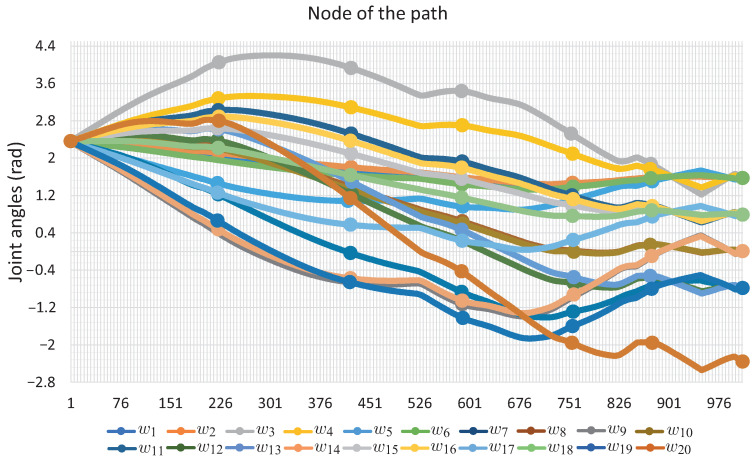
Joint *w*-angle motion for Case Study 3.

Figure 19 presents the sequence of robot arm simulation of the study case 3. In this picture, each position corresponds to a marked point in Figure 20. Figure 20 shows the evolution of the angles *w* along the path obtained for Case Study 3.

**Figure 21:**
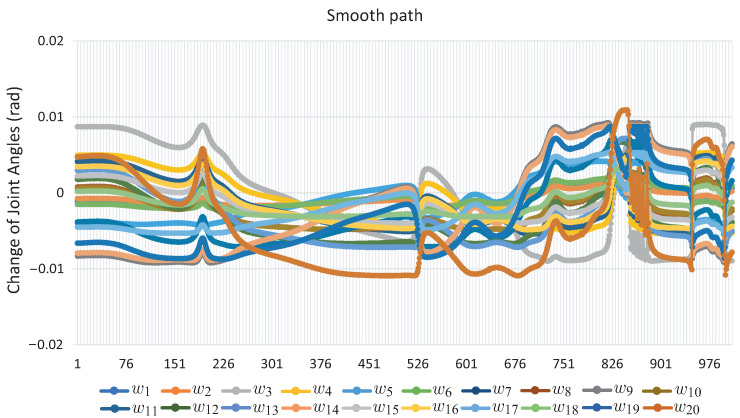
Change of joint angle motion of Case Study 3.

Figure 20 shows the evolution of the angles *w* along the path obtained for this. It can be seen that it exhibits a smooth displacement throughout the points that correspond to the images in Figure 21. The smoothness of the path can be validated by the nature of the homotopy continuation methods, which generate a continuous solution curve. Figure 21 shows the change in joint angle movement, where it is observed that the greatest change obtained between each movement does not exceed 0.02 radians for the full path, so the path is smooth.

Table 3 shows the simulation parameters. The successful implementation of the HPPM-PRA on hyper-redundant robot arms to compute a collision-free path opens the possibility for the method to be applied to various areas of science such as medicine and exploration, among others [5,10,12,13,14,44,45].

Table 4 shows the computation time, memory consumption, total number of hyperspheres used to trace the homotopy path, radius of the hyperspheres, and characteristics of the workspace (number of links, the amount and type of obstacles).

It can be seen that the computation time and memory consumption increase as the number of links and obstacles increases. The ellipsoidal obstacles have a higher computational cost than the circular obstacles. This is because the numerical problems that path tracking faces are due to the exponent of the ellipsoid formulation. The number of hyperspheres shown in Table 4 indicates the total movements made by the robotic arm to complete the task, so the proposed method obtains smooth paths for all case studies; this can be validated with Figure 11, Figure 12, Figure 16, Figure 17, Figure 20 and Figure 21. The nature of homotopy is to create smooth paths to solve the NAES; therefore, the paths obtained with the proposed method will be smooth. The radius of the hypersphere was assigned as 0.02 with the purpose of standardizing the value, but this can be changed for any case study. It is suggested to use a value between 0.001 and 0.04; the smaller the size, the greater the total number of hyperspheres to be obtained is and vice versa.

Case Study 1 was the one that obtained the best results in CPU time, hyperspheres, and memory consumption. This is because the complexity of this workspace is minor since it only has two obstacles and an arm with three links. For Case Study 2 and Case Study 3, the computation time and memory consumption increased because of the greater number of obstacles and links and the presence of ellipsoidal obstacles.

In the first two case studies, the computation time was less than 65 milliseconds and the memory consumption was less than 5 KB; this means that the proposed method can be implemented in embedded systems with limited memory. The memory consumption in all cases was less than 20 KB, demonstrating the low memory consumption of the proposed method. In contrast, the state-of-the-art hyper-redundant robotic arms, such as the one proposed in Case Study 3, require high-performance equipment with outstanding processor and memory consumption. They even use mathematical strategies to obtain favorable results [10,11,14], not to mention that, sometimes, they cannot find the path or only work with specific scenarios.

## 6. Implementation of the Proposed Method in the CRS Catalyst-5 Robot

The proposed method was validated through its implementation on a real robotic arm model, CRS CataLyst-5. The method so far only works with planar robot arms; thus, the movement of the CRS-CataLyst-5 robot was limited to two axes.

**Figure 22:**
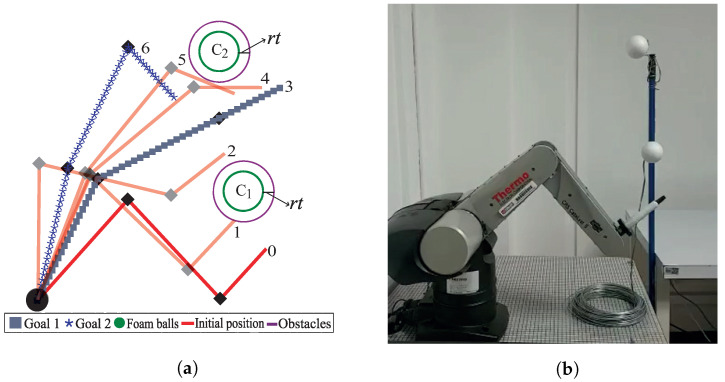
Path planning for the CRS CataLyst-5 robot. (**a**) Robot arm simulation. (**b**) Configuration of the CRS CataLyst-5 robot arm.

**Figure 23:**
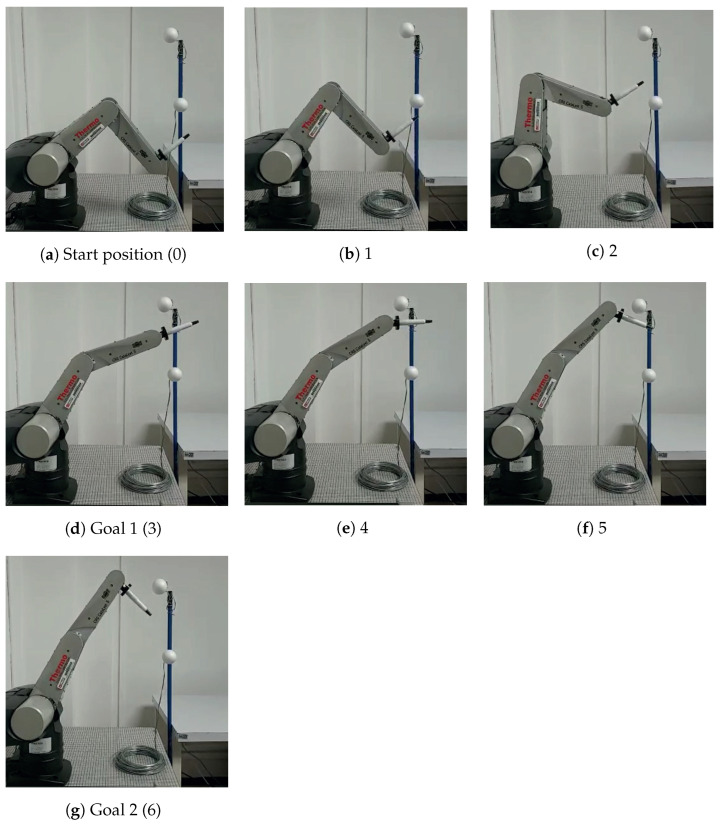
Sequence of images that describe the movement of the CRS CataLyst-5 robot avoiding two obstacles.

To carry out the implementation, the test was divided into two stages:

1. First, the workspace to be solved was established. A three-link robot arm with normalized dimensions regarding the CRS CataLyst-5 robot was used. The circular obstacles had a tolerance radius rt, which guaranteed no collision of the robot arm with the obstacle (foam balls). For this case, two goals (goal1 and goal2) were set and are depicted in Figure 22 by the line formed by gray boxes and the blue line formed by asterisks, respectively. The movements of the robot arm are semi-transparent. The robot arm first reaches Goal 1 (the first homotopic path has been followed). Then, the endpoint of this path is used as the starting position to obtain the second homotopic path and reach Goal 2. In this way, a single path is obtained capable of avoiding obstacles and meeting both goals. The computation time and memory consumption were 2 milliseconds and 0.924 KB, respectively. The sequence of movements is executed by the robotic arm, as shown in Figure 22a.
2. The second stage of this process is to adjust the numeric homotopy path data to the correct instructions for the CRS CataLyst-5 arm to follow the path. The CRS-CataLyst-5 robot has five degrees of freedom, a teach pendant, and a controller for interpreting and processing the instructions sent by the computer through its Robcomm3 software to generate the movements of the robot [46,47]. Figure 22b depicts the robotic arm workspace. From Figure 23 (implementation), the sequence of movements of Figure 22a (simulation) is corroborated.

## 7. Conclusions and Future Work

In this work, a novel method for collision-free path planning for robotic arms using homotopy continuation methods was presented. This proposal is flexible since it is capable of working in the simulation with different characteristics of the robotic arm, such as different link lengths, an arbitrary number of links, or adding grippers. Moreover, the homotopy path-planning method for planar robotic arms (HPPM-PRA) can work with circular and ellipsoidal obstacles. Obstacle avoidance is achieved by strategically adding mathematical singular points to the links. The behavior of the evasion is controlled by the repulsion parameter assigned to each obstacle, which allows obtaining a path that approaches or moves away from each obstacle when searching for the goal position.

The HPPM-PRA was tested in three different scenarios to validate the capability of the method to work with different robot arms. The results of the case studies showed 20 KB of maximum memory expended in the implementations. These results validate the low memory consumption of the HPPM-PRA. A remarkable result of the HPPM-PRA is that it can reach a goal while avoiding obstacles and crossing narrow corridors using low computational resources, which is a complex task for probabilistic methods. The HPPM-PRA method was validated with a CRS CataLyst-5 robot for practical implementations. For this, it was necessary to consider a normalized workspace and the limitations of the software and hardware of the CRS CataLyst-5 robot.

The HPPM-PRA represents a novel proposal for the homotopy continuation methods [22,23,24,35,36,37,38,39,40,41,42,43,48,49] because it faces the problem of path planning by representing the configuration space (C-space) of the robot arm by using a system of algebraic equations and strategically allocated singularities, which are fundamental during the process of circumventing the obstacles. The proof of concept proved to be effective for implementation on real robots and prepares the way for implementation on scrolling robots in 3D configuration spaces. However, the extension of this project to 3D environments is left as future work, since the representation of the configuration space must be modified in the HPPM formulation by using the Denavit–Hartenberg representation.

## Figures and Tables

**Table 1:** Parameters of Case Study 1.

Obstacle	Type of Obstacle	$x_{c}$	$y_{c}$	$r_{c}$	*P*
$C_{1}$	Circular	2.4	2.5	0.58	−0.1
$C_{2}$	Circular	1.6	3.5	0.58	0.1
Link length	$L_{1}=\sqrt{5}$, $L_{2}=\sqrt{2}$, $L_{3}=\sqrt{0.5}$				
Constants of auxiliary equations	$l_{1}:\left(a_{0}=-5.03,a_{1}=1,a_{2}=3,a_{3}=2\right)$, $l_{2}:\left(a_{0}=-3.46,a_{1}=1,a_{2}=-1,a_{3}=4\right)$, $l_{3}:\left(a_{0}=-0.32,a_{1}=1,a_{2}=1,a_{3}=-2\right)$				
Initial state of the robot arm links	$L_{1}:\left\{\left(0,0\right),\left(2,1\right)\right\}$, $L_{2}:\left\{\left(2,1\right),\left(3,0\right)\right\},L_{3}:\left\{\left(3,0\right),\left(3.5,0.5\right)\right\}$				
Final state of the robot arm links	$L_{1}:\left\{\left(0,0\right),\left(1,2\right)\right\}$, $L_{2}:\left\{\left(1,2\right),\left(2,3\right)\right\},L_{3}:\left\{\left(2,3\right),\left(2.5,3.5\right)\right\}$				

**Table 2:** Parameters of Case Study 2.

Obstacle	Type of Obstacle	$x_{c}$	$y_{c}$	$r_{c}$	$\alpha_{c}$	$\beta_{c}$	*P*
$C_{1}$	Circular	4.0	6.5	0.2	-	-	−0.0002
$E_{1}$	Ellipsoid	4.5	5.5	-	15.0	0.05	−0.8
$E_{2}$	Ellipsoid	4.5	7.5	-	15.0	0.05	0.8
Link length	$L_{1-6}=\sqrt{2}$, $L_{gripper}=\sqrt{0.5}$						
Constants of auxiliary equations	$l_{1}:(a_{0}=-13.05,a_{1}=1,a_{2}=1,a_{3}=2,a_{4}=3,a_{5}=4,a_{6}=5,$ $a_{7}=5,a_{8}=4)$, $l_{2}:(a_{0}=-6.26,a_{1}=1,a_{2}=-1,$$a_{3}=2,a_{4}=-3,a_{5}=4,a_{6}=-5,$ $a_{7}=5,a_{8}=-4)$, $l_{3}:(a_{0}=9.19,a_{1}=1,a_{2}=-1,a_{3}=-2,a_{4}=-3,a_{5}=-4,a_{6}=-5,$ $a_{7}=5,a_{8}=4)$, $l_{4}:(a_{0}=-5.73,a_{1}=1,a_{2}=3,a_{3}=-2,a_{4}=-3,a_{5}=1,a_{6}=3,$ $a_{7}=7,a_{8}=-3)$, $l_{5}:(a_{0}=-21.87,a_{1}=1,a_{2}=7,a_{3}=2,a_{4}=-3,a_{5}=6,a_{6}=7,$ $a_{7}=8,a_{8}=1)$, $l_{6}:(a_{0}=0.00,a_{1}=1,a_{2}=-1,a_{3}=-7,a_{4}=7,a_{5}=4,a_{6}=3,$ $a_{7}=1,a_{8}=-4)$, $l_{7}:(a_{0}=-13.88,a_{1}=1,a_{2}=8,a_{3}=4,a_{4}=-3,a_{5}=-3,a_{6}=-6,$ $a_{7}=6,a_{8}=8)$, $l_{8}:(a_{0}=-20.24,a_{1}=1,a_{2}=10,a_{3}=1,a_{4}=-3,a_{5}=-4,a_{6}=10,$ $a_{7}=6,a_{8}=-8)$						
Initial state of the robot arm links	$L_{1}:\left\{\left(0,0\right),\left(1,1\right)\right\}$, $L_{2}:\left\{\left(1,1\right),\left(2,2\right)\right\}$, $L_{3}:\left\{\left(2,2\right),\left(3,3\right)\right\}$, $L_{4}:\left\{\left(3,3\right),\left(4,2\right)\right\}$, $L_{5}:\left\{\left(4,2\right),\left(5,1\right)\right\}$, $L_{6}:\left\{\left(5,1\right),\left(6,0\right)\right\}$, $L_{7}:\left\{\left(6,0\right),\left(6.5,0.5\right)\right\}$, $L_{8}:\left\{\left(6,0\right),\left(6.5,0.5\right)\right\}$						
Final state of the robot arm links	$L_{1}:\left\{\left(0,0\right),\left(0,\sqrt{2}\right)\right\}$, $L_{2}:\left\{\left(0,\sqrt{2}\right),\left(0,2\sqrt{2}\right)\right\}$, $L_{3}:\left\{\left(0,2\sqrt{2}\right),\left(0,3\sqrt{2}\right)\right\}$, $L_{4}:\left\{\left(0,3\sqrt{2}\right),\left(1,5.24\right)\right\}$, $L_{5}:\left\{\left(1,5.24\right),\left(2,6.24\right)\right\}$, $L_{6}:\left\{\left(2,6.24\right),\left(3.39,6.5\right)\right\}$, $L_{7}:\left\{\left(3.39,6.5\right),\left(4.05,6.75\right)\right\}$, $L_{8}:\left\{\left(3.39,6.5\right),\left(4.05,6.25\right)\right\}$						

**Table 3:** Parameters of Case Study 3.

Obstacle	Type of Obstacle	$x_{c}$	$y_{c}$	$r_{c}$	$\alpha_{c}$	$\beta_{c}$	*P*
$C_{1}$	Circular	6.84	4.75	0.15	-	-	0.0000001
$C_{2}$	Circular	−3.0	8.0	0.5	-	-	−1.2
$E_{1}$	Ellipsoid	4.5	7.5	-	300.0	0.01	450.0
$E_{2}$	Ellipsoid	4.5	5.9	-	0.02	15.0	60.0
$E_{3}$	Ellipsoid	8.5	2.1	-	0.02	15.0	−0.0001
$E_{4}$	Ellipsoid	4.5	0.5	-	300.0	0.01	10.0
$E_{5}$	Ellipsoid	0.5	1.7	-	0.02	8.0	−63.0
Link length	$L_{1-20}=\sqrt{0.5}$						
Constants of auxiliary equations	$l_{1}:(a_{0}=-42.41,a_{1}=1,a_{2}=1,a_{3}=2,a_{4}=3,a_{5}=4,a_{6}=5,$ $a_{7}=5$, $a_{8}=4,a_{9}=3,a_{10}=2,a_{11}=1,a_{12}=-5,a_{13}=-4,$ $a_{14}=-3,$$a_{15}=-2$, $a_{16}=-1,a_{17}=-1,a_{18}=-2,a_{19}=-3,$ $a_{20}=-4)$, . . ., $l_{20}:(a_{0}=18.84,a_{1}=1,a_{2}=-1,a_{3}=9,a_{4}=7,a_{5}=-2,a_{6}=1,$ $a_{7}=-5$, $a_{8}=-4,a_{9}=-3,a_{10}=6,a_{11}=-1,a_{12}=5,a_{13}=3,$ $a_{14}=-6,$$a_{15}=-8$, $a_{16}=1,a_{17}=-10,a_{18}=2,a_{19}=3,a_{20}=8)$						
Initial state of the robot arm links	$L_{1}:\left(-0.5,0.5\right)$, $L_{2}:\left(-1,1\right)$, $L_{3}:\left(-1.5,1.5\right)$, $L_{4}:\left(-2,2\right)$, $L_{5}:\left(-2.5,2.5\right)$, $L_{6}:\left(-3,3\right)$, $L_{7}:\left(-3.5,3.5\right)$, $L_{8}:\left(-4,4\right)$, $L_{9}:\left(-4.5,4.5\right)$, $L_{10}:\left(-5,5\right)$, $L_{11}:\left(-5.5,5.5\right)$, $L_{12}:\left(-6,6\right)$, $L_{13}:\left(-6.5,6.5\right)$, $L_{14}:\left(-7,7\right)$, $L_{15}:\left(-7.5,7.5\right)$, $L_{16}:\left(-8,8\right)$, $L_{17}:\left(-8.5,8.5\right)$, $L_{18}:\left(-9,9\right)$, $L_{19}:\left(-9.5,9.5\right)$, $L_{20}:\left(-10,10\right)$						
Final state of the robot arm links	$L_{1}:\left(0,\sqrt{2}\right)$, $L_{2}:\left(0,2\sqrt{2}\right)$, $L_{3}:\left(0,3\sqrt{2}\right)$, $L_{4}:\left(0,4\sqrt{2}\right)$, $L_{5}:\left(0,5\sqrt{2}\right)$, $L_{6}:\left(0,6\sqrt{2}\right)$, $L_{7}:\left(0.5,4.74\right)$, $L_{8}:\left(1.2,4.74\right)$, $L_{9}:\left(1.91,4.74\right)$, $L_{10}:\left(2.62,4.74\right)$, $L_{11}:\left(3.12,4.24\right)$, $L_{12}:\left(3.62,3.74\right)$, $L_{13}:\left(4.12,3.24\right)$, $L_{14}:\left(4.82,3.24\right)$, $L_{15}:\left(5.32,3.74\right)$, $L_{16}:\left(5.82,4.24\right)$, $L_{17}:\left(6.32,4.74\right)$, $L_{18}:\left(6.82,5.24\right)$, $L_{19}:\left(7.32,4.74\right)$, $L_{20}:\left(6.82,4.24\right)$						

**Table 4:** Results obtained in the three case studies carried out.

Study Case	Time	Memory	Hyperspheres	Hypersphere Radius	Number of Links	Circular Obstacle	Ellipsoid Obstacle
1	3.3 ms	1.404 KB	146	0.02	3	2	-
2	61.1 ms	4.308 KB	323	0.02	8	1	2
3	2.71 s	18.272 KB	1012	0.02	20	2	5

## Data Availability

Not applicable.
